# AAGCN: a graph convolutional neural network with adaptive feature and topology learning

**DOI:** 10.1038/s41598-024-60598-2

**Published:** 2024-05-02

**Authors:** Bin Wang, Bodong Cai, Jinfang Sheng, Wenzhe Jiao

**Affiliations:** School of Computer Science and Engineering, Central South University, Changsha, 410000 China

**Keywords:** Deep learning, Complex network, Graph convolutional network, Applied mathematics, Computer science

## Abstract

In recent years, there has been a growing prevalence of deep learning in various domains, owing to advancements in information technology and computing power. Graph neural network methods within deep learning have shown remarkable capabilities in processing graph-structured data, such as social networks and traffic networks. As a result, they have garnered significant attention from researchers.However, real-world data often face challenges like data sparsity and missing labels, which can hinder the performance and generalization ability of graph convolutional neural networks. To overcome these challenges, our research aims to effectively extract the hidden features and topological information of graph convolutional neural networks. We propose an innovative model called Adaptive Feature and Topology Graph Convolutional Neural Network (AAGCN). By incorporating an adaptive layer, our model preprocesses the data and integrates the hidden features and topological information with the original data’s features and structure. These fused features are then utilized in the convolutional layer for training, significantly enhancing the expressive power of graph convolutional neural networks.To evaluate the effectiveness of the adaptive layer in the AAGCN model, we conducted node classification experiments on real datasets. The results validate its ability to address data sparsity and improve the classification performance of graph convolutional neural networks.In conclusion, our research primarily focuses on addressing data sparsity and missing labels in graph convolutional neural networks. The proposed AAGCN model, which incorporates an adaptive layer, effectively extracts hidden features and topological information, thereby enhancing the expressive power and classification performance of these networks.

## Introduction

Graph neural networks are a type of deep learning models specifically designed for graph-structured data. They have the capability to handle various types of graph data, such as social networks and protein-protein interaction (PPI) networks. By leveraging graph neural networks, it becomes possible to model and predict information related to nodes, edges, and graphs themselves, enabling tasks like node classification, link prediction, and community detection.

The application of graph neural networks extends to several practical engineering fields, including recommendation systems^1^, biomedicine^2^, traffic prediction^3^, and natural language processing^4^. These networks have demonstrated strong engineering application value and have been successfully utilized in various domains.

Early graph neural network models primarily relied on graph convolution operations, which extended the convolution operations from traditional convolutional neural networks to graphs, effectively extracting and learning features of nodes and edges. However, these early models had limitations, such as the inability to handle graphs with arbitrary shapes and poor performance on large-scale graphs.

As graph neural networks continue to evolve, new research directions have emerged. For instance, there is an increasing focus on applying graph neural networks to small-sample learning^5^, reinforcement learning^6^, and transfer learning^7^. Researchers have also explored the application of graph neural networks in heterogeneous graphs^8^, dynamic graphs^9^, and combinatorial optimization^10^. To meet practical application needs, researchers constantly propose new models and algorithms, aiming to improve the interpretability and generalization ability of graph neural networks.

Graph convolutional neural networks, as a prominent representative of graph neural networks, serve as powerful tools for data analysis and modeling, capable of solving problems that traditional deep learning models struggle with. They remain an active area of research with significant value. However, graph data often presents challenges such as data sparsity^11^, feature sparsity^12^, and missing labels^13^, which hinder the practical application of graph convolutional neural networks.

To overcome these challenges, we propose an innovative model called Adaptive Feature and Topology Graph Convolutional Neural Network (AAGCN), which builds upon the original graph convolutional neural network research. AAGCN addresses the feature sparsity issue by employing a one-dimensional convolution layer to adaptively process the feature matrix in graph data. Additionally, it tackles the incomplete expression of topological information in graph data by defining a two-dimensional node embedding matrix to adaptively mine potential topological relationships.

This article starts by introducing relevant symbols and definitions in the “Preliminaries” section, laying the foundation for the entire work. The core idea of the algorithm is then explained, showcasing the integration of adaptive feature matrix calculation and adjacency matrix method with the original graph convolutional neural network into the AAGCN model. The effectiveness of the AAGCN model is validated through node classification experiments conducted on three real-world citation networks: Cora, PubMed, and Citeseer.

## Related work

Graph convolutional neural networks (GCNs) are a powerful class of deep learning algorithms specifically designed for processing graph data. They leverage the topological relationships and attribute information between nodes and edges to learn node and edge representations, which can be applied to various tasks such as node classification, graph classification, node clustering, and link prediction.

The development of GCNs can be traced back to traditional graph algorithms like PageRank^14^, community detection, and shortest path. With the advancements in deep learning and computing capabilities, GCNs have experienced rapid progress in recent years. In this section, we will introduce several representative GCN models.

ChebNet: ChebNet is a spectral-based GCN model that approximates graph convolution operations using Chebyshev polynomials^15^. This approach enables the extraction and learning of node and edge features in graph data. ChebNet is capable of handling graphs with arbitrary shapes and learning variable graph convolution filters through training the weights of Chebyshev basis functions. It has demonstrated good performance on real-world datasets.

GCN (Graph Convolutional Network): GCN is a semi-supervised learning method that operates directly on graph-structured data^16^. It employs a localized first-order approximation of spectral graph convolutions, similar to ChebNet but with greater simplicity and efficiency. GCN effectively extracts and learns features from nodes and edges in graph data, achieving higher classification accuracy compared to ChebNet on citation networks such as Cora, PubMed, and Citeseer.

GraphSAGE (Graph Sample and Aggregate): GCN training requires access to the entire graph structure information, which can be computationally expensive. GraphSAGE addresses this issue by using a multi-layer aggregation method^17^. It is a graph convolutional neural network that combines sampling and feature aggregation, improving computational efficiency by sampling and aggregating neighbor features. GraphSAGE captures both local and global information of nodes and can be trained using supervised or unsupervised learning methods. It achieves powerful inductive learning capabilities through parameter sharing.

GAT (Graph Attention Network): GAT is a graph convolutional neural network that incorporates the attention mechanism^18^. It calculates the similarity between each node and its neighbors to determine their importance and performs weighted summation on the neighbor nodes. The core idea of the GAT model is based on attention mechanism, assigning different attention weights to different neighbor nodes to construct embedding vectors. GAT can adapt to the relative importance of nodes and their neighbors, effectively handling complex graph structures, and achieving excellent results in various graph applications.

Despite their strengths, these methods have certain limitations in fully mining hidden features or expressing features adequately. Therefore, there is a need to enhance the ability to extract hidden features and topological information in graph data.

## Preliminaries

### Fundamentals

Graph convolution is indeed a specialized type of convolution designed specifically for processing graph data. Graph data typically consists of nodes and edges, where each node represents an object and each edge represents a relationship between two nodes.

In graph convolution, the features of each node and its neighboring nodes are convolved to produce a new feature representation. This operation takes into account the connection relationships between nodes and combines the features of each node with those of its neighbors. A learnable parameter matrix, known as the convolution kernel, is used to perform a weighted sum of the features of the node and its neighboring nodes. This process effectively generates new node features that incorporate information from the surrounding nodes.

By leveraging graph convolution, we can capture the structural dependencies and relationships present in graph data, enabling us to extract meaningful features that can be utilized for various tasks such as node classification, link prediction, and community detection. The ability of graph convolution to consider the connections between nodes and combine their features makes it a powerful tool for analyzing and modeling graph-structured data.

From a mathematical point of view, graph convolution can be seen as a filtering operation on the signal on the graph. Suppose there is a graph $G = (V,E)$.Given an *n*-dimensional vector signal $x \in R^n$, where *n* represents the number of nodes in the graph. Graph convolution operation can be seen as a linear transformation of the signal *x*, that is $y = \Theta x$, where $\Theta$ is a learnable filter matrix. In graph convolution operation, $\Theta$ is usually a sparse matrix, whose each row corresponds to a node in the graph, and each column corresponds to a neighbor node in the graph. Suppose the set of neighbor nodes of node $v_i$ is *N*(*i*), then the element $\Theta _{i,j}$ of the *i*-th row and *j*-th column of $\Theta$ represents the weight between node $v_i$ and neighbor node $v_j$. Therefore, as shown in equation 1, for node $v_i$, its corresponding output signal $y_i$ can be calculated as:

1 $$\begin{aligned} y_i = \sum _{j\in N(i)} \Theta _{i,j}x_j \end{aligned}$$

This computation process can be seen as a weighted sum of the signals of the neighbor nodes of node $v_i$, where the weights are the corresponding elements in the filter matrix $\Theta$. Finally, the output signals $y \in R^n$ of all nodes can be represented as a signal processed by the filter $\Theta$. Specifically, in graph convolutional neural networks, the graph convolution of the $l+1$-th layer can be expressed as equation 2:

2 $$\begin{aligned} H^{(l+1)} = \sigma (\tilde{D}^{-\frac{1}{2}}\tilde{A}\tilde{D}^{-\frac{1}{2}}H^{(l)}W^{(l)}) \end{aligned}$$

where $H^{(l)}$ represents the feature representation of the nodes in the *l*-th layer, $W^{(l)}$ is the weight matrix of the *l*-th layer, $\sigma$ is the activation function, $\tilde{A}$ is the adjacency matrix *A* plus self-loops, and $\tilde{D}$ is the degree matrix *D* plus self-loops. The graph convolution operator can be seen as a function of the adjacency matrix, which multiplies the adjacency matrix and the feature matrix, and normalizes the result. In the normalization process, $\tilde{D}^{-\frac{1}{2}}$ normalizes the degree of nodes, and $\tilde{A}$ normalizes the adjacency matrix, thus ensuring that graph convolution has repeatability and locality properties.

### Symbols and definitions

To lay the foundation for the following work, we list the symbols and definitions used in this section, as shown in Table 1.

**Table 1:** Symbols and definitions.

Symbol	Definitions
*G*	Undirected graph *G*, composed of all sets of points *V* and edges *E*
*V*	The set of all points in graph *G*
*E*	The set of all edges in graph *G*
*X*	Feature matrix, representing the feature vectors of nodes, each row represents a feature of a node
$\hat{X}$	Feature matrix after adaptive one-dimensional convolution processing
*A*	Adjacency matrix, representing the connection relationship between nodes
$\tilde{A}$	Adjacency matrix after self-loop
$\dot{A}$	Self-loop adjacency matrix after adaptive two-dimensional node embedding matrix processing
$\hat{A}$	Adaptive adjacency matrix after row and column quadratic scaling by degree matrix
*D*	Degree matrix, representing the degree of each node
$\tilde{D}$	Degree matrix corresponding to the adjacency matrix after self-loop
*H*	Hidden layer feature matrix, the matrix composed of all node features
*W*	Weight matrix, the weight of node features in convolution
$\sigma$	Activation function, the nonlinear function applied to the output of each layer
$\Theta$	Graph convolution filter matrix
$\odot$	Hadamard product, the multiplication of corresponding positions of two matrices
*Emb*	Two-dimensional node embedding matrix dimension
$CONV_{1d}$	One-dimensional convolution lawyer
*Z*	Represents the output of the AAGCN model
$v_i$	The *i*th node

## Graph Convolutional Neural Network based on Adaptive feature and topology

### Proposed model

**Figure 1:**
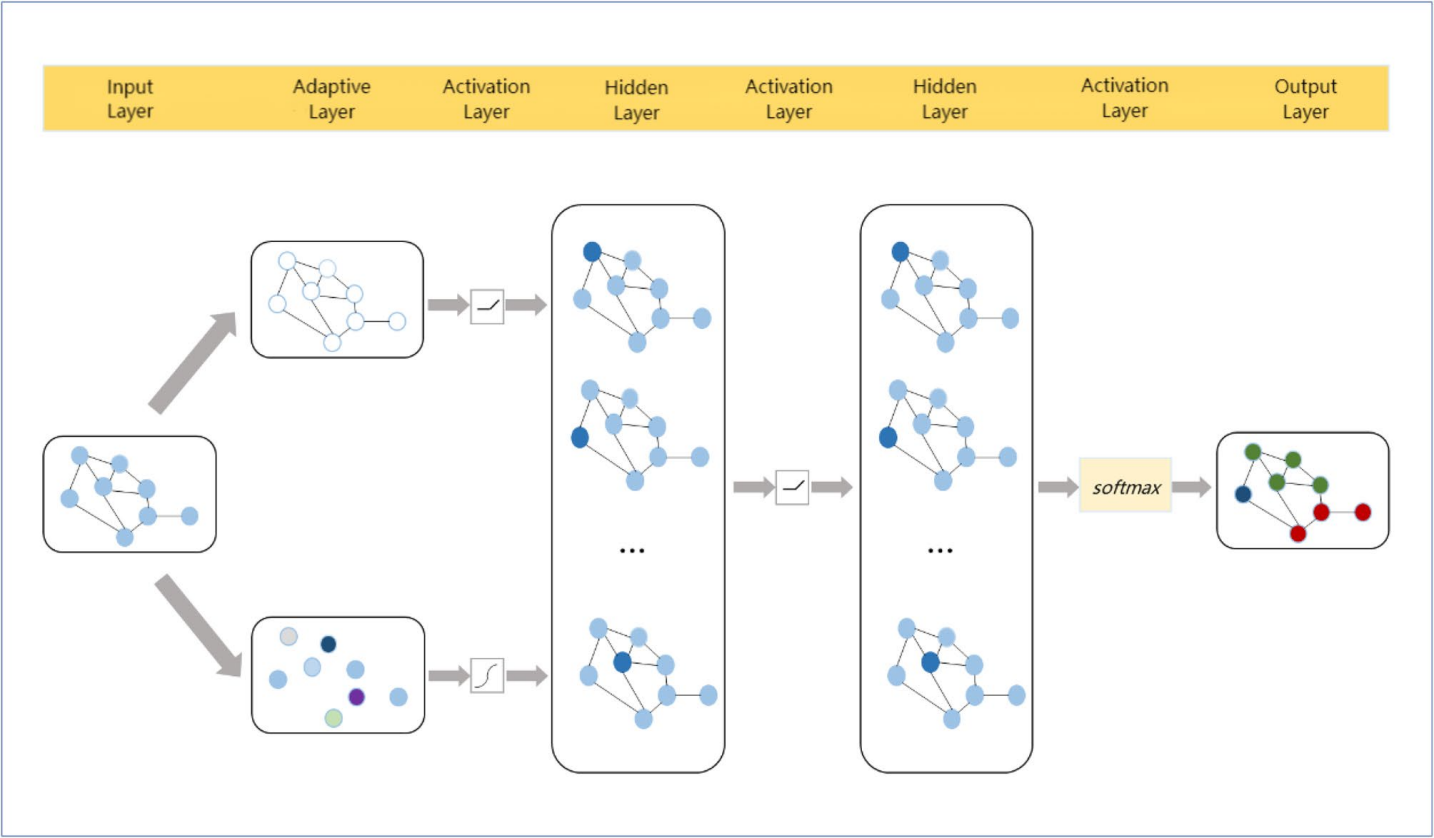
AAGCN model architecture diagram.

In this section, we will provide a detailed explanation of the AAGCN algorithm, which stands for Adaptive Features and Topology based Graph Convolutional Neural Network. The core idea of AAGCN is to dynamically adjust the feature matrix and adjacency matrix based on different graph structures and task requirements to extract the most relevant and useful information.

**Algorithm 1:**
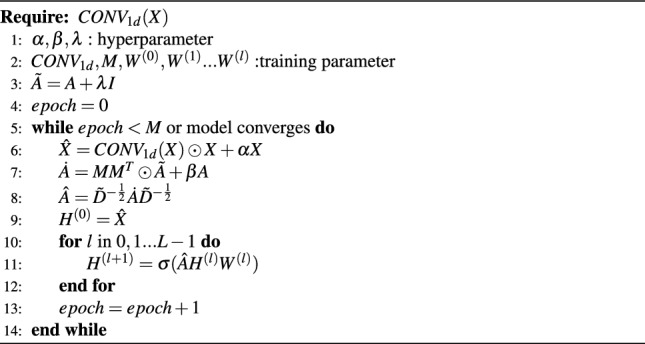
AAGCN model

The AAGCN model is an end-to-end model, as depicted in Fig. 1. The training process involves several steps. First, the graph data is preprocessed and passed into the AAGCN model through the input layer. The feature matrix is processed using one-dimensional convolution, and the output is fused with the original feature matrix to generate a new feature matrix. This new feature matrix is then input into the convolution layer.

Simultaneously, the two-dimensional node embedding is initialized and multiplied by its transpose matrix. The result is then subjected to the Hadamard product with the adjacency matrix of the graph, generating a new adjacency matrix. This new adjacency matrix is also input into the convolution layer.

The hidden layer of the model uses activation functions such as and , and the output of the hidden layer is sent to the output layer. The output layer, which primarily utilizes the function, classifies and outputs the results. The loss function used is cross-entropy loss, and the model parameters are updated through backpropagation. The training process continues until the specified number of training rounds is reached or the model converges and ends early.

The training algorithm for the AAGCN model is presented in Algorithm 1. This algorithm outlines the steps involved in training the model and updating its parameters.

### Model derivation

The AAGCN model first preprocesses the undirected graph $G = (V,E)$ as shown in equation 3, where $V = v_1,v_2,...,v_n$ represents the node set, the edge $(v_i,v_j) \in A$ between nodes $v_i$ and $v_j$, $A_{ij}$ represents the edge weight between nodes $v_i$ and $v_j$, in this paper, if there is an edge between nodes $v_i$ and $v_j$, then $A_{ij} = 1$, otherwise 0. Given an input feature matrix $X \in R^{N \times S}$, where *S* represents the feature dimension, *N* is the number of nodes, $X_{i}$ represents the feature vector of node $v_{i}$. An adaptive two-dimensional node embedding matrix *M* is defined, which has a shape of $N \times Emb$, where *Emb* is the embedding dimension.

3 $$\begin{aligned} A \in R^{N \times N} \nonumber \\ X \in R^{N \times S} \nonumber \\ M \in R^{N \times Emb} \nonumber \\ D_{ii} = \sum _{j \ne i} A_{ij} \end{aligned}$$

This paper uses a one-dimensional convolutional layer with a kernel size of 5 to process the feature matrix *X*, to mine the potential information in the feature matrix. As shown in equation 4, $\hat{X}$ is used to represent the feature matrix *X* and the feature matrix *X* processed by the one-dimensional convolutional layer as the feature input of the AAGCN model, where $\alpha$ is a controllable hyperparameter, and $\odot$ is the Hadamard product.

4 $$\begin{aligned} \hat{X} = CONV_{1d}(X) \odot X + \alpha X \end{aligned}$$

We use *AX* to represent the aggregation process of obtaining feature representations from neighbor nodes, that is, the graph convolution process. Since only the feature aggregation of neighbor nodes is considered in the process and self-features are ignored, in equation 5, $\tilde{A}$ represents the adjacency matrix *A* plus the self-loop after the adjacency matrix, where *I* is the identity matrix and $\lambda$ is 1, and $\tilde{D}$ represents the degree matrix of $\tilde{A}$.

5 $$\begin{aligned} \tilde{A} = A + \lambda I \end{aligned}$$

This paper uses the adaptive two-dimensional node embedding matrix *M* to multiply its transpose matrix $M^{T}$ on the left, and obtains an $N \times N$ matrix with the same shape as the original adjacency matrix $\tilde{A}$. As shown in equation 6, $\dot{A}$ represents the original self-looped adjacency matrix $\tilde{A}$ and the adaptive two-dimensional node embedding matrix $MM^{T}$ as the spatial information input of the model, where $\beta$ is a controllable hyperparameter.

6 $$\begin{aligned} \dot{A} = MM^T \odot \tilde{A} + \beta A \end{aligned}$$

As shown in Eq. 7, $\tilde{D}^{-\frac{1}{2}}\dot{A}\tilde{D}^{-\frac{1}{2}}$ represents the scaling of the columns and rows of the adjacency matrix $\dot{A}$ by the degree matrix $\tilde{D}$, because the aggregation process of neighbor node features may result in huge vectors for nodes with large degrees, and vice versa.

7 $$\begin{aligned} \hat{A} = \tilde{D}^{-\frac{1}{2}}\dot{A}\tilde{D}^{-\frac{1}{2}} \end{aligned}$$

Then we get Eq. 8, where $H^{(l+1)}$ represents the output of the $l+1$-th convolutional layer, and when $l = 0$, $H^{(0)}$ is the feature matrix $\hat{X}$, $W^{(l)}$ is the weight matrix of the *l*-th layer, and $\sigma$ is the activation function.

8 $$\begin{aligned} W^{(l)} \in R^{F^{(l-1)} \times F^{(l)}} \nonumber \\ H^{(l+1)} = \sigma (\hat{A}H^{(l)}W^{(l)}) \end{aligned}$$

The output of the AAGCN model with two convolutional layers can be represented by *Z* in the equation 9 , where *ReLU* is the activation function of the convolutional layer, and *softmax* is the activation function of the output layer. First, the graph *G* is sent to the adaptive layer of the model, and after being processed by the adaptive layer, the feature matrix *X* and the adjacency matrix *A* are sent to two hidden layers with *ReLU* as the activation function, and finally output the classification result through *softmax* activation function.

9 $$\begin{aligned} Z = f(X,A) = softmax(\hat{A}ReLU(\hat{A}\hat{X}W^{(0)})W^{(1)}) \end{aligned}$$

## Experiments

### Datasets

We have conducted experimental verification on three real-world datasets to evaluate the performance of the AAGCN model. As shown in Table 2, these datasets are Cora, Citeseer, and PubMed, all of which are citation network datasets.

**Table 2:** Summary of datasets.

Dataset	Nodes	Edges	classes	Features
Cora	2708	5429	7	1433
PubMed	19,717	44,338	3	500
Citeseer	3327	4732	6	3703

The Cora dataset (https://linqs-data.soe.ucsc.edu/public/lbc/cora.tgz) consists of 2708 papers from seven different disciplines, including case-based, genetic algorithms, neural networks, probabilistic methods, reinforcement learning, rule learning, and theory. Each paper in the Cora dataset has 1433 words as features, and there are 5429 edges representing the citation relationships between the papers. The citation network in the Cora dataset exhibits characteristics of a small-world network, with a high clustering coefficient and short path length.

The Citeseer dataset (https://linqs-data.soe.ucsc.edu/public/lbc/citeseer.tgz) is also composed of academic papers and contains 3,312 papers from six different disciplines. Each paper in the Citeseer dataset has 3,703 words as features, and there are 10,543 edges representing the citation relationships between the papers.

The PubMed dataset (https://linqs-data.soe.ucsc.edu/public/Pubmed-Diabetes.tgz), on the other hand, is a literature dataset in the medical field. It consists of 19,717 papers, with each paper having 500 features. There are 232,386 edges representing the citation relationships between the papers. Similar to the Cora dataset, the PubMed citation network is also a small-world network, where each node represents a medical paper and each edge represents the citation relationship between two papers.

These three datasets, Cora, Citeseer, and PubMed, are widely used in practical applications and serve as fundamental datasets for evaluating various graph neural network algorithms. Researchers can utilize these datasets to assess and compare the performance of different algorithms, providing a foundation and guidance for addressing more complex graph machine learning problems.

### Evaluation metrics

We use ACC (Accuracy) as the evaluation metric to measure the performance of the model in node classification experiments. The calculation process is shown in equation 10.

10 $$\begin{aligned} \textrm{ACC}=\frac{\sum _{i=1}^N\zeta \left( pre(v_i),tru(v_i)\right) }{N} \nonumber \\ \zeta (x, y) = {\left\{ \begin{array}{ll} 1, x = y \\ 0, x \ne y \end{array}\right. } \end{aligned}$$

Where $pre(v_i)$ represents the predicted label of node $v_i$, $tru(v_i)$ represents the true label of node $v_i$, $\zeta (x,y)$ represents the comparison result of predicted label and true label, and the larger the value of *ACC*, the better the node classification performance.

In addition to ACC, we also introduce the Macro-F1 score as another metric to evaluate the performance of the model in node classification experiments. The Macro-F1 score is the average of the F1 scores for all categories, taking both the precision and recall of the model into account, which is particularly useful for datasets with imbalanced classes. The calculation process for Macro-F1 is shown in equation 11:

11 $$\begin{aligned} \mathrm{Macro-F1}=\frac{1}{N}\sum _{i=1}^{N}\frac{TP_{i}}{TP_{i}+\frac{1}{2}(FP_{i}+FN_{i})} \end{aligned}$$

Here, $TP_{i}$ denotes the number of true positive predictions for category *i*, where the model correctly identifies positive instances. $FP_{i}$ represents the number of false positive predictions for category *i*, where the model incorrectly labels negative instances as positive. $FN_{i}$ signifies the number of false negative predictions for category *i*, where the model fails to recognize positive instances. *N* is the total number of categories. The Macro-F1 score provides a way to measure the model’s overall performance across all categories, particularly emphasizing the balance between precision and recall. This metric is especially valuable in scenarios where class imbalances might skew the accuracy metric, as it equally weighs each class’s contribution, regardless of its size.

### Baseline methods

We conduct node classification experiments on Cora, Citeseer and PubMed datasets with the same settings as GCN, and compare the experimental results with the mainstream node classification methods. The baseline methods are as follows:

ManiReg^19^: This method is a semi-supervised learning method based on manifold regularization (Manifold Regularization), which regularizes the feature vectors of each node in the manifold space to capture the structural information between nodes, and learns a node classifier on this basis.

SemiEmb^20^: This method is a semi-supervised graph embedding (Semi-supervised Embedding) method, which is used to learn low-dimensional representations of nodes in graphs. Its basic idea is to use labeled data to constrain the representations of unlabeled data, while minimizing the differences between nodes in the embedding space.

LP^21^: This method is a semi-supervised learning method based on label propagation (Label Propagation), which aims to label the unlabeled nodes in the graph. The basic idea of the method is to use the label information of known nodes, and infer the labels of unknown nodes through the similarity between nodes.

DeepWalk^22^: This method is a network embedding method based on random walk, which aims to map the nodes in the network to vector representations in low-dimensional space. The method generates node sequences by randomly walking in the network, and uses these sequences as training data for learning.

ICA^23^: This method is a machine learning method for label classification tasks by iterative classification (Iterative Classification). The working principle of ICA is to iteratively train a classifier on a subset of labels, then use the classifier to predict the labels of the remaining instances, then use the predicted labels to update the subset of labels, and repeat this process until convergence.

Planetoid^24^: This method is a semi-supervised learning method based on embedding, which uses the topology structure of the graph as a form of regularization, and learns node embedding by jointly predicting the class labels and neighborhood contexts of the nodes in the graph.

GraphSAGE^17^: This method is a node representation learning method based on graph neural network, which mainly samples and aggregates information from each node’s neighbors. It can generate low-dimensional vector representations for each node in the graph, with strong scalability and generality.

GAT^18^: This method uses attention mechanism to learn the relationships between nodes, and uses multi-head attention mechanism on each node. It can learn the weights between each node and its adjacent nodes, and multiply them by feature vectors to produce output vectors for each node.

GCN^16^: This method is a deep learning model based on graph structure data, which can learn node features by extending convolution operations to graphs. Its core idea is to use adjacency matrix and node feature matrix to perform node information aggregation, where adjacency matrix represents the connection relationship between nodes, and node feature matrix represents the attribute features of each node.

### Experimental results

The experimental environment for this study was configured as follows: In terms of hardware, the system utilized an Intel(R) Xeon(R) Gold 6230R CPU @ 2.10GHz as the central processing unit, paired with an NVIDIA-RTX3090 graphics processing unit. It was equipped with 128GB of system memory and 24GB of video memory. On the software front, experiments were conducted on an Ubuntu 18.04 operating system, using Python 3.8.16 as the programming language. Model development and training were facilitated by the PyTorch 1.9.1 deep learning framework, which supports CUDA 11.7.99 to fully leverage the computational capabilities of the NVIDIA-RTX3090 GPU.

#### Comparison experiment

The experimental results presented in this paper are based on the average of 50 repeated experiments. The results of the baseline algorithms are obtained using their optimal parameters. The hyperparameters used in the experiments are as follows: the dimension of the convolutional layer is set to 32, the dropout rate is 0.78, the learning rate is 0.005, the training is performed for 200 epochs, the kernel size for one-dimensional convolution is 5, the dimension of the two-dimensional node embedding matrix is 64, and the hyperparameters $\alpha$ and $\beta$ are set to 0.6 and 0.2, respectively. However, for the PubMed dataset, the dimension of the convolutional layer is set to 16, and the dimension of the two-dimensional node embedding matrix is set to 32.

**Figure 2:**
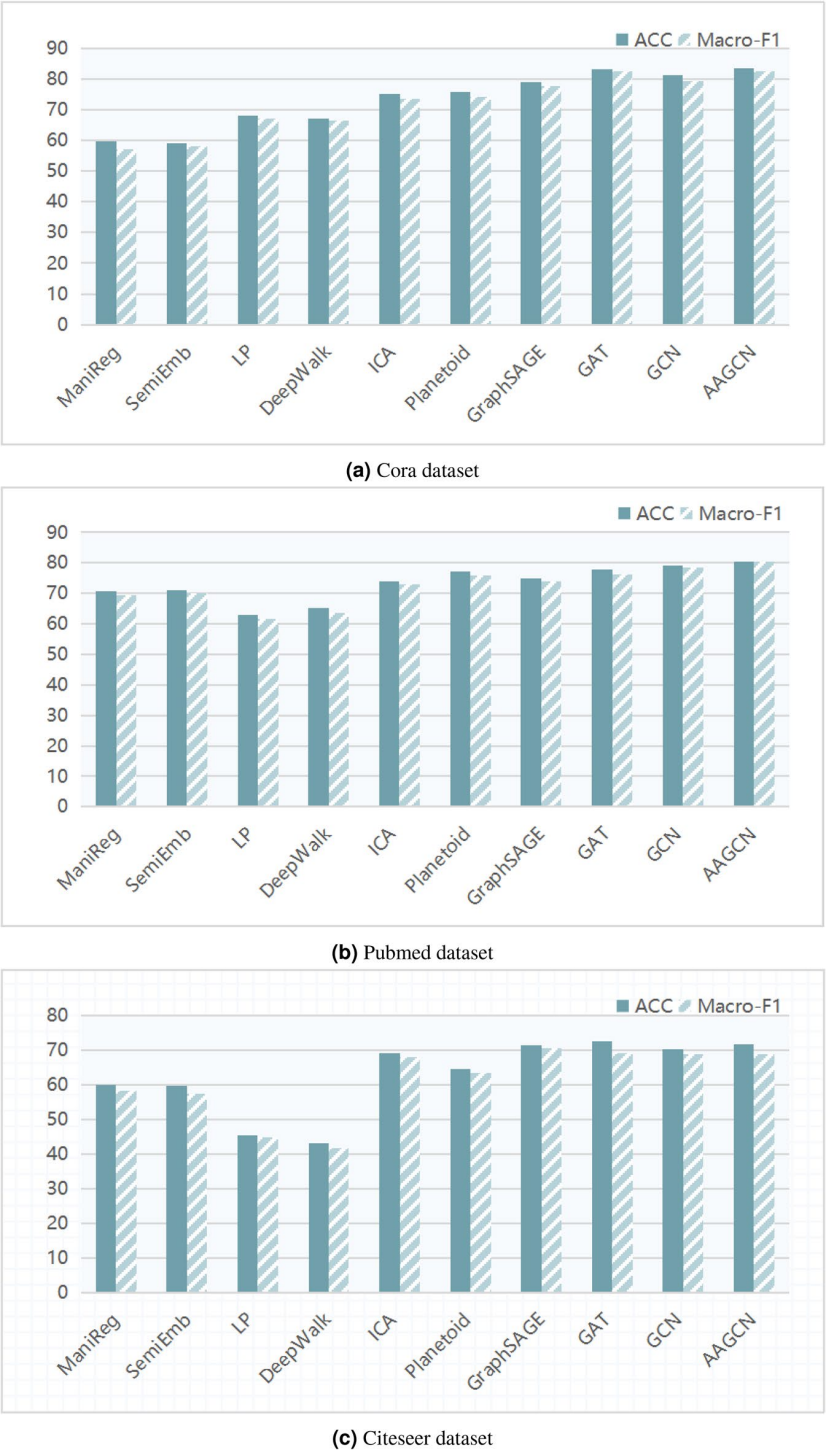
Node classification performance comparison of different methods on Cora, Pubmed and Citeseer datasets.

**Figure 3:**
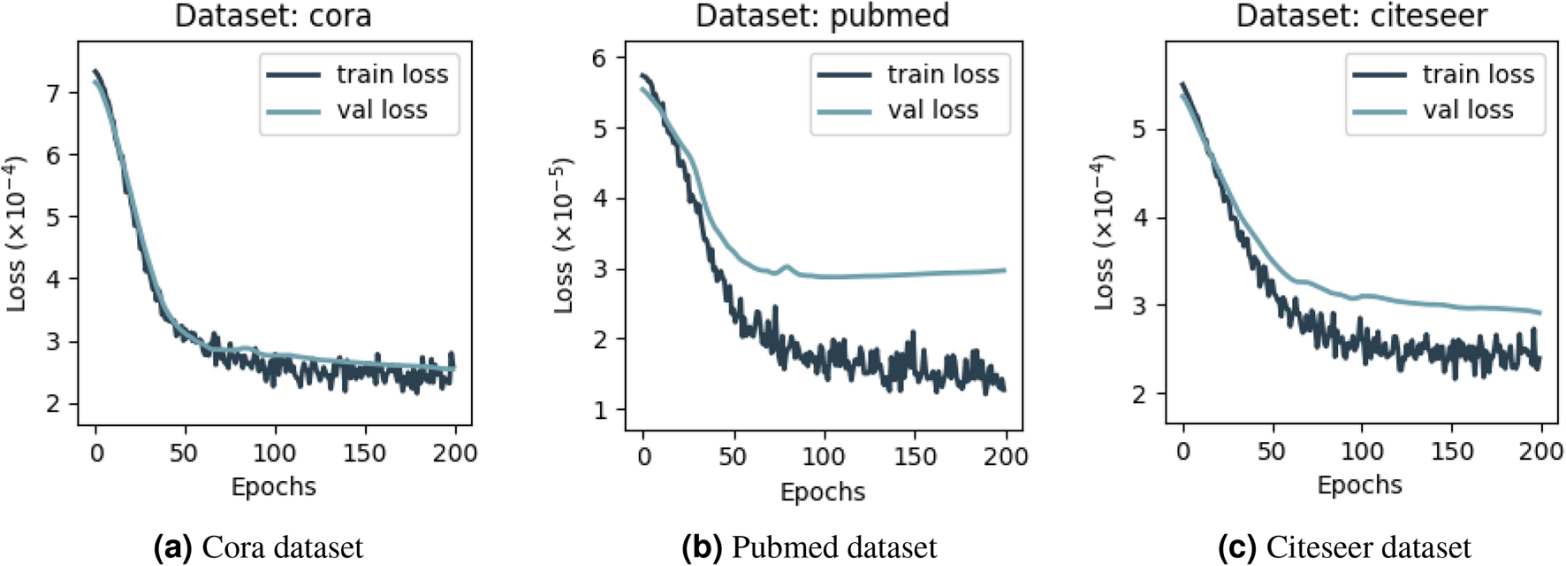
Comparison of AAGCN Model’s Training and Testing Loss over 200 Epochs on Cora, PubMed, and Citeseer Datasets.

Table 3 presents the results of node classification experiments on the three real datasets. The best results are highlighted in black bold, and the second best results are underlined.

**Table 3:** Node classification experimental results of various methods on datasets.

Method	Cora		Pubmed		Citeseer	
	ACC	Macro-F1	ACC	Macro-F1	ACC	Macro-F1
ManiReg	59.5	57.2	70.7	69.4	60.1	58.4
SemiEmb	59.0	58.1	71.1	69.9	59.6	57.3
LP	68.0	67.1	63.0	61.7	45.3	44.7
DeepWalk	67.2	66.5	65.3	63.6	43.2	41.6
ICA	75.1	73.7	73.9	73.1	69.1	67.9
Planetoid	75.7	74.4	77.2	75.8	64.7	63.4
GraphSAGE	78.9	77.8	75.0	74.0	71.6	70.7
GAT	83.0	82.4	77.8	76.3	**72.5**	**69.1**
GCN	81.3	79.4	79.0	77.8	70.3	68.9
AAGCN	**83.3**	**82.5**	**80.4**	**80.2**	71.6	69.0

From Table 3, it can be observed that the AAGCN model outperforms the classic GCN model in node classification experiments on the Cora, PubMed, and Citeseer datasets. In comparing the performance of the AAGCN model with existing models, we observe that on the Cora dataset, AAGCN achieves a classification accuracy of 83.3% and a Macro-F1 score of 82.5, closely matching the GAT model and significantly outperforming other methods. On the PubMed dataset, the AAGCN model leads with the highest accuracy of 80.4% and a top Macro-F1 score of 80.2, demonstrating its powerful capability in handling graph-structured data on large-scale datasets. On the Citeseer dataset, AAGCN maintains commendable performance with an accuracy of 71.8% and a Macro-F1 score of 69.1, despite the GAT model showing a slight advantage on this dataset. Overall, the AAGCN model demonstrates its robustness and adaptability across all three datasets, particularly excelling in handling class balance issues, underscoring its potential application and research value in the field of graph neural networks.

Figure 2 illustrates the node classification results of different methods on the three real datasets. It can be observed that the AAGCN model, with its adaptive feature matrix and adjacency matrix, improves the node classification performance of the original GCN model. It successfully addresses the challenges of the node feature coefficient problem and the hidden topological information mining problem in the original graph data. In order to further demonstrate the superiority of the AAGCN model, we will separately discuss the node classification performance when using only the adaptive feature matrix or the adaptive adjacency matrix on the three datasets. We will also investigate the influence of the number of convolutional layers on the experimental performance.

In Fig. 3, we present the training and validation loss curves for our model on the Cora, Citeseer, and PubMed datasets. These curves illustrate the loss trajectory over 200 epochs, indicating the model’s learning progress and generalization capabilities.

For the Cora dataset, the training loss demonstrates a steep decline in the initial epochs, indicating rapid learning, and then plateaus, suggesting the model’s convergence. The validation loss closely tracks the training loss, initially, and then stabilizes, reflecting the model’s good generalization to unseen data. The Citeseer dataset shows a similar pattern, with both training and validation losses quickly declining and then flattening out. The consistent gap between the training and validation loss curves suggests the model is well-fitted without significant overfitting. For the PubMed dataset, we observe an early and pronounced decrease in training loss, with a gentle yet consistent decrease in validation loss thereafter. The validation loss for this dataset demonstrates less volatility and a smoother decline, indicating a robust fitting process despite the complexity of the data.

Across all three datasets, the convergence of the validation loss with the training loss, without significant divergence, underscores the model’s ability to generalize across different graph-structured tasks. This is evidence of the AAGCN model’s efficacy and stability during the training process and its adeptness at learning from graph-structured data.

#### Ablation Experiment

The main focus of this experiment is to investigate the impact of the adaptive feature module (AAGCN-X) and the adaptive topology module (AAGCN-A) of the AAGCN model on improving the node classification performance of the traditional GCN model. Node classification experiments are conducted on the three real-world datasets mentioned earlier, using the parameter settings described in the previous section.

**Table 4:** The ablation experiment results of different methods on the datasets.

Method	Cora	PubMed	Citeseer
GCN	81.3	79.0	70.3
AAGCN-X	**83.8**	79.2	71.0
AAGCN-A	82.4	78.7	71.3
AAGCN	83.3	**80.4**	**71.6**

**Figure 4:**
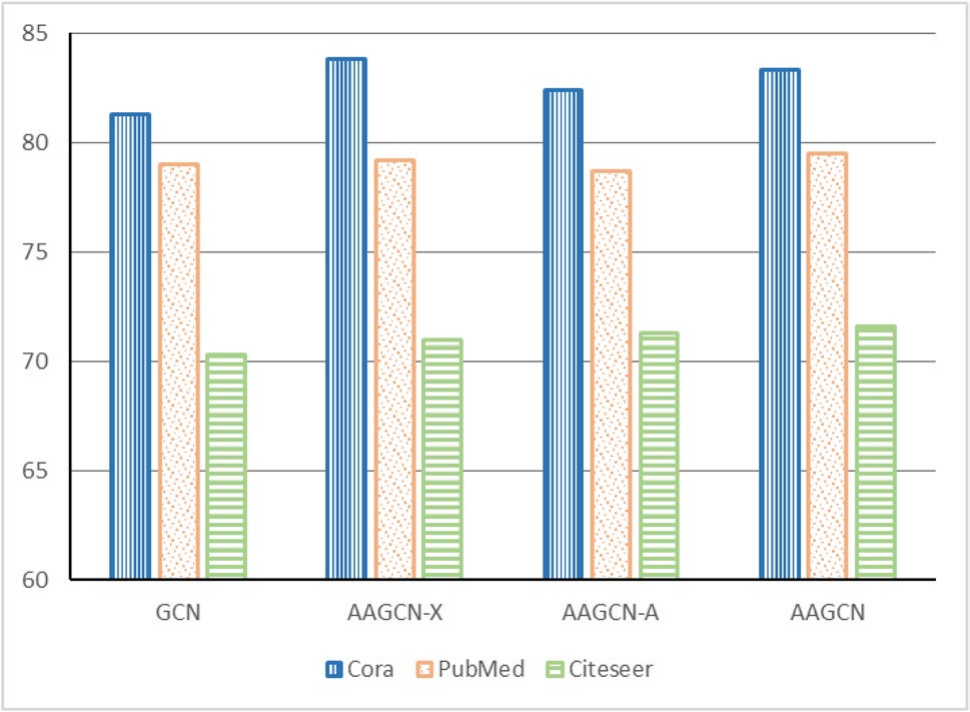
Effect diagram of node classification in ablation experiment.

Table 4 presents the results of these experiments, which are similar to the interpretation of Table 3. From the table, it can be observed that the AAGCN-X model achieves the best performance on the Cora dataset, outperforming the GCN model by 2.5%. Except for the AAGCN-A model, which performs slightly lower than the GCN model by 0.3% on the PubMed dataset, all other models achieve higher accuracy than the GCN model on the node classification results of the three real-world datasets. Comparatively, the AAGCN model performs slightly lower than the AAGCN-X model by 0.5% on the Cora dataset, slightly higher by 1.2% on the PubMed dataset, and higher by 0.6% on the Citeseer dataset.

From Fig. 4, it can be observed that the AAGCN model consistently exhibits better node classification performance compared to the other three models. This further confirms the effectiveness and reasonableness of combining the adaptive feature module and the adaptive topology module of the AAGCN model in enhancing the node classification performance of the traditional GCN model.

#### Experiment on Model Layers

**Table 5:** The experimental results with different layers on the datasets.

Layers	Cora	PubMed	Citeseer
Layer-1	74.7	73.2	67.9
Layer-2	**83.1**	**80.4**	**71.5**
Layer-3	81.3	76.6	68.1
Layer-4	80.3	73.9	62.5
Layer-5	78.6	73.3	58.5
Layer-6	77.6	75.2	61.9
Layer-7	75.2	73.9	61.3
Layer-8	74.3	72.3	58.9
Layer-9	74.7	74.7	57.4
Layer-10	74.9	73.5	55.7

The objective of this experiment is to examine the performance of the AAGCN model with varying numbers of stacked convolutional layers on real-world datasets. Using the same parameters, we conducted 10 experiments and obtained the average results. We tested the AAGCN model with one to ten convolutional layers stacked, and the results are presented in Table 5.

**Figure 5:**
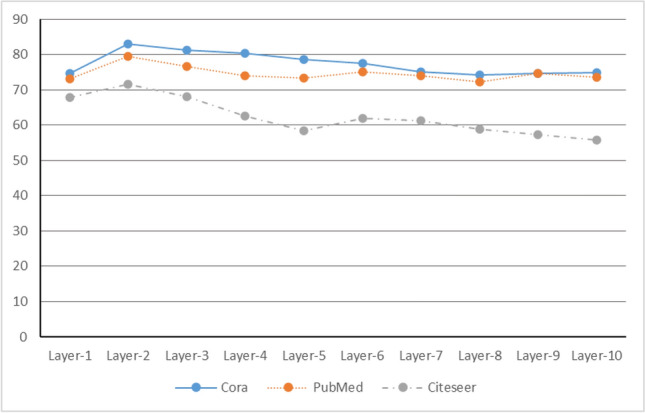
The change of node classification performance of AAGCN with different layers, when the layer is 2, the performance is optimal.

From Fig. 5, it can be observed that the node classification experiment results are the best when the AAGCN model has a stack depth of two layers on all three datasets. However, as the depth increases beyond two layers, the accuracy of node classification decreases. This phenomenon can be attributed to over-smoothing, where the increasing number of convolution layers leads to the convergence of node features in the network, resulting in reduced discriminative power. Consequently, we determine that the optimal depth for the convolutional layers in the AAGCN model is two layers.

Therefore, based on the experimental results, we conclude that the AAGCN model with two convolutional layers achieves the best node classification performance on the tested datasets.

#### Experiment on Hyperparameter

**Figure 6:**
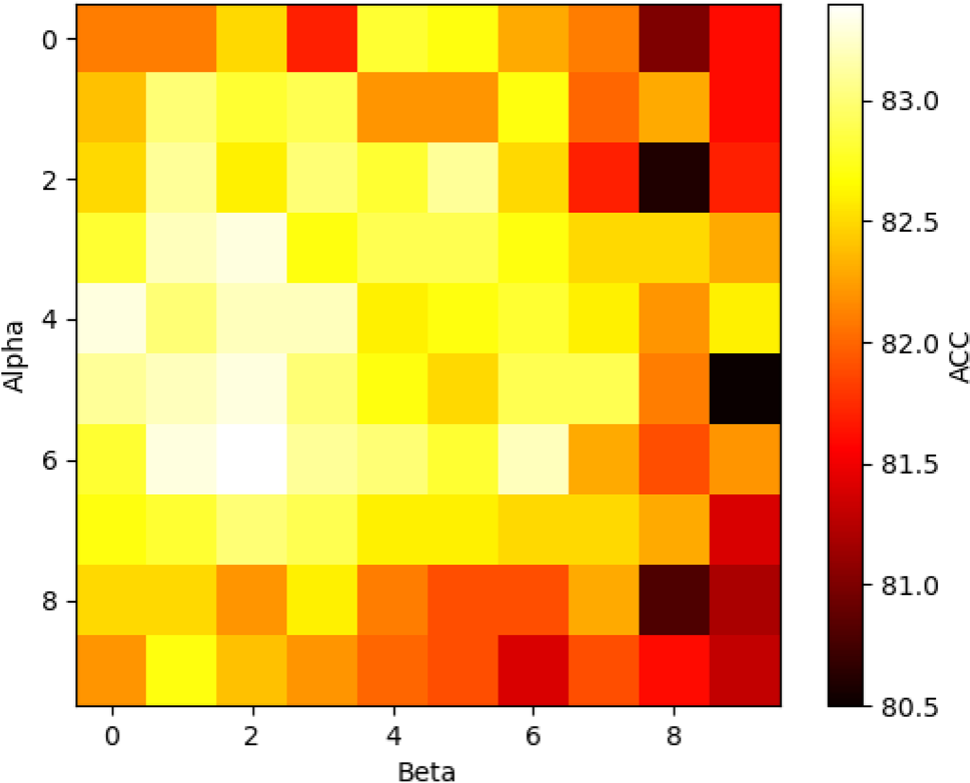
The performance of AAGCN on Cora dataset under different hyperparameters, where the lighter the color, the higher the accuracy of the hyperparameter.

The objective of this experiment is to evaluate the node classification performance of the AAGCN model on real-world datasets by varying the values of the hyperparameters $\alpha$ and $\beta$. Taking the Cora dataset as an example, we conducted 10 experiments and obtained the average results with a stacking layer number of 2 for the AAGCN model.

From Fig. 6, it can be observed that when the hyperparameters $\alpha$ and $\beta$ are set to 0, the AAGCN model can be considered equivalent to the original GCN model. However, as we gradually increase the values of $\alpha$ and $\beta$ from 0 to 0.7, which introduces the adaptive layer, the AAGCN model improves the node classification performance compared to the GCN model. Notably, when the hyperparameter $\alpha$ is approximately 0.6 and $\beta$ is approximately 0.2, the AAGCN model exhibits the highest node classification performance. Consequently, we set the hyperparameters $\alpha$ and $\beta$ in the AAGCN model to 0.6 and 0.2, respectively.

Based on these findings, we conclude that introducing the adaptive layer with appropriate values of $\alpha$ and $\beta$ can enhance the node classification ability of the AAGCN model. Therefore, we select $\alpha = 0.6$ and $\beta = 0.2$ as the optimal values for these hyperparameters in the AAGCN model.

## Conclusion

In this paper, we propose an adaptive feature and topology based graph convolutional neural network model (AAGCN). Given a graph, the model adaptively processes the feature matrix through a one-dimensional convolution layer, and the adjacency matrix through a two-dimensional node embedding matrix, and jointly inputs them into the convolution layer, to mine the potential feature and topology information of graph data. The model uses cross entropy as the loss function, and updates the model parameters through back propagation. After deriving the computational principle of the model, we conduct node classification experiments on three real citation network datasets Cora, PubMed and Citeseer, and use accuracy as the evaluation metric. By comparing with the baseline methods, and conducting experiments on different depths and hyperparameters of the AAGCN model, we fully demonstrate the effectiveness and rationality of the AAGCN model and hyperparameter selection.

However, there are still some improvements that can be made to the model proposed in this paper. Although the model proposed in this paper improves the original GCN’s ability to mine hidden node features and topology information, in the adaptive layer, the dimension of the two-dimensional node embedding matrix is , which increases the time and space complexity of the algorithm to some extent. Therefore, proposing a lightweight adaptive module becomes the focus of the next work. In addition, the model proposed in this paper requires to send the global structure of graph data into the model for training, which makes it not suitable for large-scale network learning training. In future work, we consider integrating aggregation operations similar to spatial convolution into this model.

## Data Availability

The data that support the findings of this study are available on request from the corresponding author, J.S., upon reasonable request.
